# 超高效合相色谱法快速测定保健食品中10种脂溶性维生素

**DOI:** 10.3724/SP.J.1123.2022.02010

**Published:** 2022-12-08

**Authors:** Jiachen LI, Ling CAO, Fang FANG, Haiwei SHI, Qing HUANG, Li TAN, Qiaolian DUAN, Youlong FENG

**Affiliations:** 1.南京中医药大学药学院, 江苏 南京 210023; 1. College of Pharmacy, Nanjing University of Chinese Medicine, Nanjing 210023, China; 2.江苏省食品药品监督检验研究院, 江苏 南京 210019; 2. Jiangsu Institute for Food and Drug Control, Nanjing 210019, China

**Keywords:** 超高效合相色谱, 脂溶性维生素, 保健食品, ultra performance convergence chromatography (UPC^2^), fat-soluble vitamins, health foods

## Abstract

脂溶性维生素作为保健食品重要的功效指标,现有的标准方法存在测定组分少、样品前处理过程复杂、对人员操作能力要求较高等问题,因此建立一种快速、简便、准确,且能同时检测多种常见脂溶性维生素的方法具有重要的现实意义。该研究采用超高效合相色谱(UPC^2^)建立了同时测定保健食品中维生素A棕榈酸酯、维生素A醋酸酯、维生素E醋酸酯、维生素K_1_、*α*-生育酚、*β*-生育酚、*γ*-生育酚、*δ*-生育酚、维生素D_2_、维生素D_3_等10种常用脂溶性维生素的方法。样品经含75%二甲基亚砜(DMSO)的水溶液在45 ℃水浴超声15 min破乳,再加入正己烷振摇提取90 min, 3000 r/min离心10 min,取上清液过滤后进行分析。使用LC-Simulator软件对色谱条件进行模拟及优化,选用ACQUITY UPC^2^ Viridis HSS C18 SB色谱柱进行分离,CO_2_和乙腈-甲醇(85∶15, v/v)为流动相,梯度洗脱,流速1.9 mL/min,柱温30 ℃,选取10种脂溶性维生素各自的最大吸收波长检测,外标法进行定量。结果表明,10种脂溶性维生素在各自范围内线性关系良好,相关系数(*r*)均大于0.9997,检出限(LOD)和定量限(LOQ)分别为片剂:0.2~30 μg/g和0.8~75 μg/g,胶囊:0.4~60 μg/g和2~150 μg/g,样品平均加标回收率在96.5%~113.9%之间,RSD均小于4%,精密度、稳定性、重复性测定结果的RSD值也均小于2%。经比较,该方法测定结果与现有的国家食品安全标准基本一致,但该方法简单、快速、灵敏、准确,可满足保健食品中10种脂溶性维生素的检测要求,为保健食品中脂溶性维生素的快速同时检测奠定基础。

## Abstract (en)

Fat-soluble vitamins are important efficacy indicators in health foods because they are essential for human physiological functions. The existing method for the simultaneous determination of fat-soluble vitamins has various problems, such as limited determination components, complex sample, pretreatment process, and high requirements for personnel operating ability. Therefore, establishing a fast, simple, and accurate method that can detect various common fat-soluble vitamins at the same time is necessary. In this study, a method for the simultaneous determination of 10 commonly used fat-soluble vitamins such as vitamin A acetate (VA acetate), vitamin A palmitate (VA palmitate), vitamin E acetate (VE acetate), vitamin K_1_ (VK_1_), *α*-tocopherol, *β*-tocopherol, *γ*-tocopherol, *δ*-tocopherol, vitamin D_2_(VD_2_) and vitamin D_3_ (VD_3_) in health foods was established by ultra performance convergence chromatography (UPC^2^). First, the contents of about 1.0 g of capsule samples were accurately weighed. A grinder was used to grind tablet samples into powder. The powder mixture was then precisely weighed at 2.0 g. Both substances were placed in 50 mL brown stopper tubes. The test tube was then filled with 20 mL 75% dimethylsulfoxide (DMSO) aqueous solution for demulsification. The tubes were then sonicated before being extracted with *n*-hexane. The centrifuged supernatant was added to vials for detection. Viridis HSS C18 SB column (100 mm×3.0 mm, 1.8 μm) was applied and CO_2_ was used as the mobile phase A. After comparing the influence of acetonitrile, methanol, and their mixture on chromatographic peak separation, acetonitrile-methanol (85∶15, v/v) was used as the mobile phase B. The injection volume was 1 μL. Using simulator software, the optimal chromatographic conditions were obtained after a set of three-factor orthogonal experiments of flow rate, gradient slope, and column temperature. The flow rate and column temperature were both set at 1.9 mL/min and 30 ℃. Furthermore, the maximum absorption wavelength of these 10 fat-soluble vitamins was selected for detection. Ten vitamins were baseline separated after 7 min of gradient elution. The limits of detection (LODs) and quantification (LOQs) of capsule samples were 0.4-60 μg/g and 2-150 μg/g, respectively, whereas the results for tablet samples were 0.2-30 μg/g and 0.8-75 μg/g. The linear ranges of the 10 fat-soluble vitamins were 0.1-100 μg/mL. The recoveries of spiked samples ranged from 96.5% to 113.9%, with RSD values less than 4%. Precision, stability, and repeatability RSD values were all less than 2%. By comparison, the determination results of this method were basically consistent with the existing national food safety standards. This method is simple, rapid, sensitive, and accurate, and it can meet the detection requirements of the 10 fat-soluble vitamins in health foods. Simultaneously, this method lays the foundation for the rapid and simultaneous detection of fat-soluble vitamins in existing health foods.

近年来随着人民生活品质的提高,越来越多的人选择服用保健食品来补充人体所需的营养成分,其中使用较多的一类是维生素类补充剂。维生素主要包括脂溶性维生素和水溶性维生素两大类。其中,脂溶性维生素因可溶于有机溶剂,并以与脂肪相似的方式被吸收和转运而得名。常见的脂溶性维生素包括维生素A、维生素D、维生素E和维生素K。脂溶性维生素的测定方法主要有荧光法^[1]^、分光光度法^[2]^、电化学法^[3]^、气相色谱法^[4]^、高效液相色谱法^[5,6]^、柱交换色谱法^[7]^、加压毛细管电色谱法^[8]^等。

保健食品中因常使用天然而非人工合成的原料,基质较为复杂、干扰成分较多,脂溶性维生素的测定难度也大大增加。目前使用较多的方法是GB 5009.82-2016《食品安全国家标准 食品中维生素A、D、E的测定》和GB 5009.158-2016《食品安全国家标准 食品中维生素K_1_的测定》。上述标准大多采用高效液相色谱法、液相色谱-串联质谱法等对维生素A、D、E、K中的一个或几个组分进行测定,但无法对这4种维生素同时测定,且样品前处理过程复杂,对人员操作能力要求较高。国家食品药品监督管理总局补充检验方法BJS 201717《保健食品中9种脂溶性维生素的测定》采用液相色谱-串联质谱法测定9种脂溶性维生素,但未包括维生素A棕榈酸酯、*α*-生育酚、*β*-生育酚、*γ*-生育酚、*δ*-生育酚等保健食品中常见的脂溶性维生素成分。

超高效合相色谱(UPC^2^)是以超临界流体色谱法为主体,使用容易达到超临界状态的CO_2_为主要流动相,配合不同有机溶剂作为改性剂,通过与不同检测器联用,来进行分离和分析的技术^[9⇓-11]^。UPC^2^分析柱的填料颗粒极细,一般在2 μm以下,分析过程中可以通过对压力、流速等条件的精细调控,实现对待测物的有效保留和分离。该技术同时还具备有机溶剂使用量少、灵敏度高、分析速度快等优点^[10,12⇓-14]^。虽然该技术已用于脂溶性维生素^[15,16]^的分析,但是应用于基质复杂的保健食品中仍未见报道。

本研究采用超高效合相色谱法,使用LC-Simulator软件进行色谱条件优化,在7 min内实现了保健食品中10种常用脂溶性维生素的分离及含量测定。本方法快速、准确,样品前处理简便,灵敏度高,重复性好,准确度高,实用性强,为维生素定性与定量检测提供了一种高效可行的色谱检测方法。

## 1 实验部分

### 1.1 仪器、试剂与材料

ACQUITY超高效合相色谱仪(配有二极管阵列检测器以及Waters Empower TM^3^数据处理系统),美国Waters公司;RF-20A高效液相色谱仪,日本Shimadzu公司;ST-16R冷冻离心机,美国Thermo Scientific公司;Tube-mill 100控制型试管研磨机,美国IKA公司。

维生素A棕榈酸酯(批号:100295-201801,纯度:98.5%)、维生素D_2_(批号:100155-201908,纯度:99.8%)、维生素D_3_(批号:100061-202110,纯度:100%)、维生素E醋酸酯(批号:100062-201811,纯度:98.0%)、维生素K_1_(批号:100156-201806,纯度:99.7%),中国食品药品检定研究院;*α*-生育酚(批号:LRAB6618,纯度:100%)、*γ*-生育酚(批号:LRAB6135,纯度:100%)、*δ*-生育酚(批号:LRAB6619,纯度:100%),美国Sigma-Aldrich公司;维生素A醋酸酯(批号:G131856,纯度:98.5%),德国Dr. Ehrenstorfer公司;*β*-生育酚(批号:8-KPA-128-4,纯度:95%),加拿大Toronto Research Chemicals公司;乙腈、甲醇、正己烷、二甲基亚砜均为色谱纯,德国Merck公司;甲酸,色谱纯,美国ACS公司;0.22 μm滤膜,上海安谱科技股份有限公司。

样品A(剂型:胶囊,批号:9624569-07,成分:天然维生素E醋酸酯)、样品B(剂型:胶囊,批号:817030801,成分:天然*α*-生育酚)、样品C(剂型:片剂,批号:1818842576471,成分:维生素A醋酸酯、维生素E醋酸酯、维生素D_3_)、样品D(剂型:片剂,批号:EW8999,成分:维生素K_1_),均为自购样品。

### 1.2 溶液的制备

精确称取各标准品25 mg于10 mL容量瓶内,用适量正己烷溶解并定容至刻度,配制成质量浓度均为2500 μg/mL的标准储备液,于4 ℃下避光保存。精确移取标准储备液适量并用正己烷稀释成100.0、75.0、50.0、25.0、10.0、5.0、2.0、0.5和0.1 μg/mL的混合标准工作液。

### 1.3 样品前处理

精密称取胶囊样品内容物1.0 g(片剂样品则为碾碎的粉末混合物2.0 g)于50 mL棕色具塞试管中,加入75%二甲基亚砜水溶液20 mL,密塞,摇匀,45 ℃水浴超声15 min,并时时振摇,冷却至室温,精密加入正己烷15 mL,机械振摇90 min,转移至50 mL离心管中,3000 r/min离心10 min,取上清液,经0.22 μm滤膜过滤后,直接进样分析。

### 1.4 色谱条件

色谱柱:ACQUITY UPC^2^ Viridis HSS C18 SB (100 mm×3.0 mm, 1.8 μm),流动相:A为CO_2_, B为乙腈-甲醇(85∶15, v/v)。梯度洗脱:0~3.5 min, 2%B~6%B; 3.5~6.0 min, 6%B~15%B; 6.0~6.1 min, 15%B~2%B; 6.1~7.0 min, 2%B。流速:1.9 mL/min;进样量:1 μL;柱温:30 ℃;检测波长:325 nm(维生素A醋酸酯、维生素A棕榈酸酯)、284 nm(维生素E醋酸酯)、254 nm(维生素K_1_)、294 nm(*α*-生育酚、*β*-生育酚、*γ*-生育酚、*δ*-生育酚)、264 nm(维生素D_2_、维生素D_3_);动态背压(ABPR): 13.10 MPa;补偿泵(0.2%甲酸甲醇溶液)流速:0.3 mL/min。

## 2 结果与讨论

### 2.1 色谱条件的选择与优化

#### 2.1.1 色谱柱的选择

为使10种脂溶性维生素及其衍生物在较短时间内达到分离,并具有良好峰形,比较了ACQUITY UPLC HSS C18(100 mm×3.0 mm, 1.7 μm)和ACQUITY UPC^2^ Viridis HSS C18 SB (100 mm×3.0 mm, 1.8 μm)这两种色谱柱对10种脂溶性维生素分离的影响。虽然UPC^2^ Viridis HSS C18 SB柱和UPLC HSS C18柱都为C18柱,但是两种色谱柱填料不同,使得两种色谱柱对10种脂溶性维生素的分离产生了差别,结果表明,特别是在分离维生素D_2_、维生素D_3_时,UPC^2^ Viridis HSS C18 SB柱的分离效果明显好于UPLC HSS C18柱。

#### 2.1.2 流动相的选择

超高效合相色谱一般以CO_2_作为流动相A,以有机试剂作为流动相B。流动相B有两个作用:第一,可提高CO_2_的溶解能力;第二,可作为强洗脱溶剂。流动相B的改变会影响保留和选择性。

本实验共考察了以甲醇、乙腈以及两者不同比例的混合溶液(乙腈∶甲醇分别为95∶5、85∶15、75∶25、50∶50, v/v)作为流动相B时的情况。甲醇在超高效合相色谱中极性很强,而乙腈相比而言则极性较弱。使用甲醇作为流动相B时,10种脂溶性维生素的出峰时间虽然提前了很多,但是维生素D_2_、维生素D_3_两个峰却无法得到很好分离。而当使用乙腈作为流动相B时,由于极性较弱,部分化合物出峰时间较长,在相同梯度条件下,维生素D_2_、维生素D_3_均未出峰。因此选用二者的混合溶液作为流动相B,并对不同比例混合溶液的效果进行比较。结果如图1所示,当甲醇比例较高时各化合物出峰较快但分离不够理想,当乙腈比例较高时各化合物间的分离度较好。综合考虑各化合物的出峰时间及分离效果,筛选出二者最佳混合比例为乙腈-甲醇(85∶15, v/v),此时不仅所有化合物都能在较短的时间内出峰,各化合物也能得到有效分离。

**图1:**
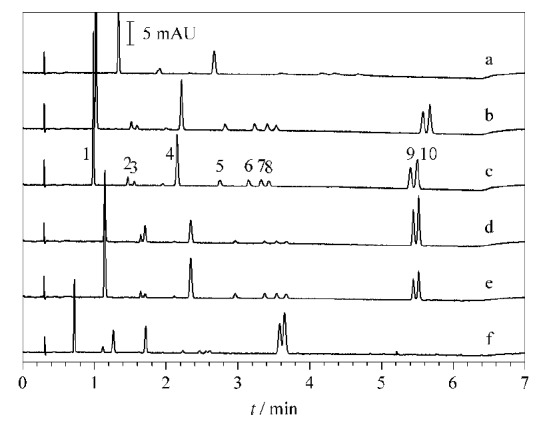
不同比例甲醇和乙腈作为流动相B时10种脂溶性维生素的色谱图

#### 2.1.3 LC-Simulator软件对色谱条件的优化

为了实现多组分化合物的分离,可以对流速、柱温、流动相组成和梯度等参数进行优化,通过人工干预进行色谱优化费时、费力,计算机辅助分离建模已逐渐成为分析化学中非常有效的工具^[17⇓⇓⇓⇓⇓-23]^。

流速以及改性剂的浓度都会影响到出峰时间,流速越快、改性剂比例越高(即极性增加),则待测物出峰越快;而柱温会影响流动相在色谱柱中的密度,随着柱温升高,流动相的密度会降低,从而延长保留时间,同时对于待测物的保留性能也有一定影响^[24,25]^。

设计以下三因素三水平进行正交试验(见表1):流速(Ⅰ)、梯度斜率(Ⅱ)和柱温(Ⅲ)。得到的27组数据经过ACD Labs软件处理,进入LC-Simulator软件进行建模,得到的三维模型见图2a,三因素正交二维模型见图2b、c、d。如图2a的3D模型显示,颜色从蓝到红的过程即为分离度从小到大变化的过程,最终选择流速1.9 mL/min,柱温30 ℃,按照该流速和柱温,调整梯度斜率,综合考虑峰位、分离度以及出峰时间之后,选择了多段式的梯度(见1.4节)。实际出峰时间与软件预测出峰时间相比,变化值(Δ值)在5%以内,说明软件预测结果可行性良好。按优化后的条件采集色谱图如图3所示。

**表1:** 正交试验的因素及水平

Level	Ⅰ(Flow rate)/ (mL/min)	Ⅱ(Gradient slope)/ (Δ%B/min)	Ⅲ(Column temperature)/ ℃
1	1.5	2.57	30
2	1.9	1.80	35
3	2.0	1.20	40

*Δ%B/min: mobile phase B ratio change rate per minute.

**图2:**
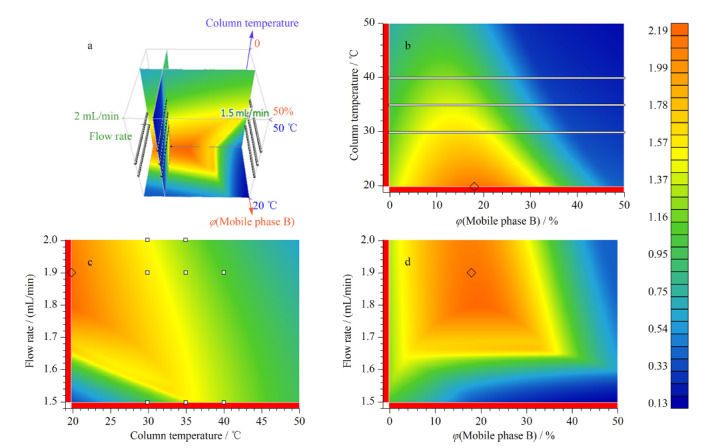
LC-Simulator软件模拟的正交试验结果

**图3:**
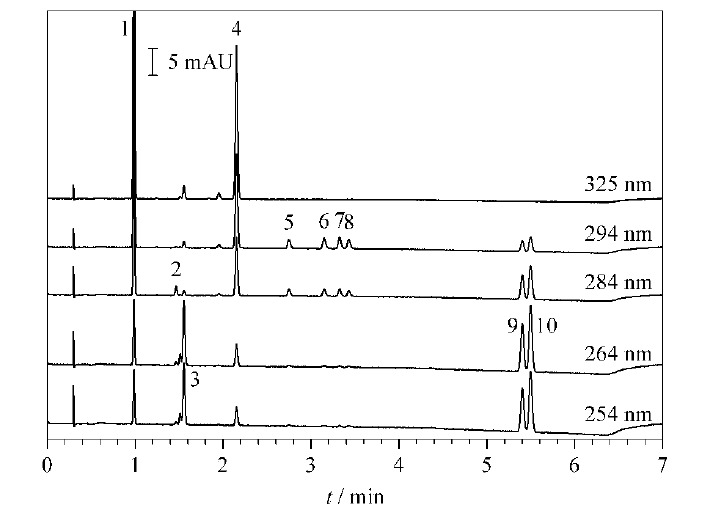
10种脂溶性维生素的超高效合相色谱图

### 2.2 前处理方法的选择

本研究对测定维生素类保健食品的前处理方法进行考察,发现正己烷可以有效地提取药品中的脂溶性维生素,但是保健食品的基质较为复杂,难以直接通过有机溶剂进行提取,特别是含维生素D的产品。经过文献^[26]^调研,发现需要先对含维生素D的样品进行破乳,才能提取完全。所以本研究最终选择先用75%二甲基亚砜水溶液破乳,再加入正己烷振荡提取作为样品的前处理方法,结果表明,经破乳后,维生素D才能被提取完全。

### 2.3 方法学考察

#### 2.3.1 线性范围、检出限及定量限

将10种脂溶性维生素系列标准工作液按1.4节色谱条件进行测定,以标准工作液质量浓度(*x*, μg/mL)为横坐标,峰面积(*y*)为纵坐标,进行线性回归分析。结果如表2所示,各化合物在线性范围内的相关系数(*r*)均大于0.9997,表示本方法线性良好。将10种脂溶性维生素系列标准工作液进行稀释,在信噪比为10(*S/N*=10)时得到10种脂溶性维生素的定量限(LOQ),在信噪比为3(*S/N*=3)时,得到10种脂溶性维生素的检出限(LOD),检出限和定量限分别为片剂0.2~30 μg/g和0.8~75 μg/g,胶囊0.4~60 μg/g和2~150 μg/g。

**表2:** 10种脂溶性维生素的回归方程、相关系数、线性范围、检出限和定量限

Vitamin	Regression equation	r	Linear range/ (μg/mL)		Tablet			Capsule	
					LOD/(μg/g)	LOQ/(μg/g)		LOD/(μg/g)	LOQ/(μg/g)
VA acetate	y=4.367×10^3^x-3.559×10^3^	0.9998	0.1-	100	0.4	0.8		0.8	2
VE acetate	y=8.575×10x+8.268	0.9999	10-	100	15	75		30	150
VK_1_	y=9.206×10^2^x-3.795×10^2^	0.9999	2-	100	4	15		8	30
VA palmitate	y=2.693×10^3^x-2.075×10^3^	0.9999	0.5-	100	0.8	4		2	8
α-Tocopherol	y=1.377×10^2^x-1.742×10^2^	0.9999	10-	100	30	75		60	150
β-Tocopherol	y=1.872×10^2^x-3.478×10^2^	0.9997	10-	100	19	75		38	150
γ-Tocopherol	y=2.299×10^2^x-2.275×10^2^	0.9999	10-	100	19	75		38	150
δ-Tocopherol	y=1.628×10^2^x-1.130×10^2^	0.9999	10-	100	19	75		38	150
VD_2_	y=1.233×10^3^x-9.426×10^2^	0.9999	2-	100	0.2	0.8		0.4	2
VD_3_	y=1.471×10^3^x-8.999×10^2^	0.9999	2-	100	0.2	0.8		0.4	2

*y*: peak area; *x*: mass concentration, μg/mL.

#### 2.3.2 精密度

在优化实验条件下,选取50 μg/mL和10 μg/mL的混合标准工作液,连续进样6次,结果表明,各组分峰面积的RSD值均小于1.3%,表示仪器精密度良好。

#### 2.3.3 稳定性

在优化实验条件下,选取供试品溶液分别在0、1、2、4、8、12、24 h进行测定。结果表明,各组分在24 h内均稳定,含量的RSD值均小于2%。

#### 2.3.4 重复性

在优化实验条件下,每份样品平行配制6份,计算含量和RSD值。结果表明,RSD均小于2%,表明该方法重复性良好。

### 2.4 样品含量测定及加标回收率

在优化实验条件下,对4种样品中维生素的含量进行了测定,同时按照国家标准GB 5009.82-2016、GB 5009.158-2016的方法进行含量测定,并进行比较(见表3)。结果表明,4种样品含量测定结果基本一致,表明建立的方法结果准确。考虑到样品中的本底值较高,对照品加入量的多少对回收率准确性影响较大。故参考《中国药典》2020年版四部通则9101分析方法验证指导原则,4种样品称样量减半分别平行配制6份,加入各样品含量50%的标准溶液,得到样品加标回收溶液,并进行加标回收率测定,结果如表3所示,4种样品平均回收率在96.5%~113.9%之间,RSD均小于4%,表明该方法准确度较高。

**表3:** 4种样品采用UPC^2^法与国家标准方法测定时含量结果比较及其加标回收率(*n*=6)

Sample	Component	Contents/(mg/g)			Added/ (mg/g)	Found/ (mg/g)	Recovery/ %	RSD/ %
		UPC^2^	GB 5009.82-2016	GB 5009.158-2016				
A	VE acetate	8.17	8.04	/	7.89	8.98	113.9	1.2
B	α-tocopherol	0.783	0.766	/	0.801	0.826	103.2	1.4
C	VA acetate	0.120	0.125	/	0.118	0.113	104.4	1.3
	VD_3_	0.00280	0.00270	/	0.00280	0.00281	100.2	2.2
	VE acetate	16.9	16.4	/	15.7	15.1	96.5	3.1
D	VK_1_	0.0370	/	0.0359	0.0349	0.0373	106.9	2.2

/: Component cannot be determined by this method.

## 3 结论

建立了同时测定保健食品中10种脂溶性维生素的超高效合相色谱方法,该方法可用于含有脂溶性维生素的保健食品的含量测定。本研究对4种含有脂溶性维生素不同剂型的保健食品进行含量测定,相比于现行脂溶性维生素的测定方法,在保证结果同样准确的前提下,体现了前处理过程简单、分析时间短的优点,有效避免了脂溶性维生素在长时间、复杂的前处理及检测过程中的损失,提高了脂溶性维生素检测的效率以及准确性。本方法可为保健食品中多种脂溶性维生素的同时测定提供技术支持,为保健食品功效成分的检测探索新的方法和思路。下一步可以考虑进一步对保健食品中其他脂溶性维生素、水溶性维生素以及黄酮、皂苷、脂肪酸等其他类别成分的检测进行探索研究,进一步拓展超高效合相色谱法在保健食品质量控制中的应用。
